# Poor sensitization of 50-kHz vocalization response to amphetamine predicts rat susceptibility to self-administration of the drug

**DOI:** 10.1007/s00213-016-4328-4

**Published:** 2016-06-02

**Authors:** Ewa Taracha, Ewelina Kaniuga, Edyta Wyszogrodzka, Adam Płaźnik, Roman Stefański, Stanisław J. Chrapusta

**Affiliations:** Department of Neurochemistry, Institute of Psychiatry and Neurology, 9 Sobieskiego St., 02-957 Warsaw, Poland; Department of Pharmacology and Physiology of the Nervous System, Institute of Psychiatry and Neurology, 9 Sobieskiego St., 02-957 Warsaw, Poland; Department of Experimental and Clinical Pharmacology, Medical University of Warsaw, 26/28 Krakowskie Przedmieście St., 00-927 Warsaw, Poland; Department of Experimental Pharmacology, Mossakowski Medical Research Centre, Polish Academy of Sciences, 5 Pawińskiego St., 02-106 Warsaw, Poland

**Keywords:** Addiction, Affective state, Amphetamine self-administration, Drug context, Drug dependence, Individual differences, Instrumental response, N-acetylcysteine, Sensitization, Ultrasonic vocalization

## Abstract

**Rationale:**

Our previous studies showed promise for using sensitization of the frequency-modulated 50-kHz vocalization response to amphetamine (AMPH) as an index of rat vulnerability to AMPH addiction.

**Objective:**

This study aimed to test the utility of sensitizing frequency-modulated (FM) 50-kHz vocalization in the AMPH self-administration paradigm as well as the ability of N-acetylcysteine to prevent self-administration relapse.

**Methods:**

Rats were subjected to the so-called two-injection protocol of sensitization (TIPS) using AMPH and were categorized as low-sensitized callers (LC_TIPS_) or high-sensitized callers (HC_TIPS_) based on the individual outcomes. Then, they were given 44 sessions of AMPH self-administration followed by a 17-session N-acetylcysteine-aided extinction course and a single session of AMPH-primed self-administration reinstatement.

**Results:**

LC_TIPS_ compared to HC_TIPS_ rats showed no considerable difference in the FM 50-kHz vocalization rate during the self-administration training or extinction course, but they were considerably more likely to acquire AMPH self-administration and experience drug-induced reinstatement of this trait. Moreover, the LC_TIPS_ rats were more likely than HC_TIPS_ rats to have a markedly higher FM 50-kHz vocalization rate after AMPH reinstatement. N-acetylcysteine did not affect the course of self-administration extinction or the instrumental or FM 50-kHz vocalization responses to AMPH reinstatement.

**Conclusions:**

There is no link between the FM 50-kHz vocalization and key characteristics of AMPH self-administration. Additionally, N-acetylcysteine does not help prevent AMPH self-administration relapse. However, there is a high predictive value for poor sensitization of the FM 50-kHz vocalization response to AMPH with respect to the acquisition and maintenance of self-administration of this psychostimulant.

## Introduction

Studies on drug addicts and corresponding animal models seem to offer the best chance for revealing the underlying neurobiological bases of addiction, which may help the development of better therapies. The transition from drug abuse to full addiction (loss of control over drug intake) seems to involve an individual vulnerability and prolonged, intensive intake of the addictive substance. This shift occurs in a varying minority (depending on the drug) of drug users, humans, and their experimental animal “equivalents” (Anthony et al. 1994; Ahmed 2012). This observation stresses the potential utility and need for early identification of susceptible individuals, which would help develop addiction prevention strategies and reduce the number of animals and costs associated with the experimental procedures aimed at developing drug dependence/addiction in laboratory rodents. However, an adequate method remains missing.

In rodent studies of addiction, increasing attention is being given to 50-kHz ultrasonic vocalization (USV), which can be induced by a variety of pleasing factors (Brudzynski and Pniak 2002; Wang et al. 2008; Brudzynski 2013; Barker et al. 2015), including stimulants and some other addictive drugs, as well as the context of prior exposure to drugs (Burgdorf et al. 2001; Ma et al. 2010; Hamed et al. 2012; Wright et al. 2012; Mahler et al. 2013; Lehner et al. 2014). Past studies on non-contingent amphetamine (AMPH) treatment (Simola et al. 2012; Taracha et al. 2012, 2014; Pereira et al. 2014) have shown that 50-kHz USV, especially the frequency-modulated (FM) variety, can reflect a number of typical drug effects, including rewarding action, sensitization, and tolerance. In addition, rats with a stronger 50-kHz USV response had stronger conditioned place preference (Burgdorf et al. 2007; Ahrens et al. 2013; Taracha et al. 2014). Apart from revealing facets of AMPH action other than conditioned place preference or locomotor stimulation (Taracha et al. 2014; Simola and Morelli 2015), sensitization of the FM 50-kHz USV response to the drug showed greater inter-individual variability and high intra-individual stability (Taracha et al. 2012). Although behavioral sensitization to repeated exposure to addictive drugs does not belong in addiction diagnosis criteria, these findings are of particular interest because they may be linked to the well-known individual differences in addiction vulnerability. As a result, these findings may open a new approach for identifying susceptible individuals in animal studies. Notably, sensitization to psychostimulants was also reported in humans and non-human primates (Strakowski and Sax 1998; Leyton 2007; Castner and Williams 2007). The existing rodent models that best represent human addiction involve self-administration of drugs. Behavioral sensitization can enhance the motivation to self-administer AMPH (Mendrek et al. 1998) and is often associated with both facilitated acquisition and escalation of stimulant self-administration (Piazza et al. 1989; Vezina 2004; Ferrario et al. 2005; but see Ball and Slane 2014), which are the initial steps for developing drug addiction in such models (Piazza and Deroche-Gamonet 2013; Deroche-Gamonet and Piazza 2014). We decided to scrutinize our findings of the varied sensitization of the FM 50-kHz USV response to AMPH (Taracha et al. 2014, 2015) by confronting them with drug self-administration-related characteristics. The main idea was to test possible predictive value of the sensitization for identifying rats with increased vulnerability to acquiring voluntary drug-taking behavior.

In drug addiction therapy, the main problem is the recurrent nature of the disorder. The present knowledge of neurochemical aberrations involved in psychostimulant drug seeking and addiction relapses indicates that the corticostriatal and corticoaccumbal glutamatergic systems have key roles (Wolf 1998; Vanderschuren and Kalivas 2000; Olive et al. 2012). Currently, there is substantial hope that the performance of these systems might be improved by pharmacological means (e.g., see Post and Kalivas 2013). One such promising agent is N-acetylcysteine (NAC), which normalizes glutamate homeostasis by increasing the expression of the GLT-1 glutamate transporter and catalytic unit of the x_c_^-^ antiporter (Kupchik et al. 2012; Lewerenz et al. 2013; Reissner et al. 2015). NAC has also shown promise for preventing relapses in rat cocaine self-administration models (e.g., see Baker et al. 2003; Amen et al. 2011; Murray et al. 2011; Frankowska et al. 2014). In our previous studies, the USV response to repeated non-contingent AMPH treatment was not modified by NAC (Taracha et al. 2015). In our present experimental design including extinction of acquired AMPH self-administration, we evaluated the potential of NAC to facilitate and stabilize this process. We examined whether (i) the varied USV response to AMPH translates into diversification of acquisition and persistence of drug self-administration and (ii) NAC can help prevent reinstatement of AMPH self-administration in a rat model. Additionally, we aimed to elucidate the meaning of FM 50-kHz USV emitted during AMPH self-administration.

## Methods and materials

### Subjects

Twenty-eight naïve male Sprague-Dawley rats from the stock of the Mossakowski Medical Research Centre were used. The rats were housed, acclimated, and habituated to experimental procedures as described earlier (Taracha et al. 2014, 2015), and weighed 315–384 g at the start of drug treatment. All experiments were performed during the light phase (7 a.m. to 7 p.m.) of the rats’ day cycle. All animal use procedures conformed to the European Communities Council Directive on the protection of laboratory animals (86/609/EEC of November 24, 1986) and to the current Polish law. The study protocol was approved by the Bioethical Committee of the Medical University of Warsaw (Certificate No. 6/2014).

### Drugs

d-Amphetamine sulfate (Sigma) was dissolved in sterile physiological saline (Sal) at required concentrations and was given to the rats either intraperitoneally, in the arenas used for testing their USV in the two-injection protocol of sensitization (TIPS; see the section on preliminary USV testing and rat categorization below), or by intravenous infusions in conditioning chambers (see the section on AMPH self-administration training below). All AMPH doses are expressed as the weight of the salt. NAC (acetylcysteine, 300 mg/3 ml; Sandoz GmbH, Austria) was injected intraperitoneally, in rats’ home cages.

### USV recording equipment and data analysis

USV calls were collected with a single model CM16 condenser microphone (Avisoft Bioacoustics, Germany) from each rat. The microphones were sensitive to frequencies of 15–180 kHz and were coupled with a custom-made amplifier of 600 Ω input impedance, 16 V/V (12 dB) voltage gain, and ±0.1 dB (30 Hz–100 kHz) frequency response. The amplified signal was passed through a custom-made anti-aliasing filter and then transferred to a PC equipped with a PCI-703-16A acquisition board (Eagle Technology, Eagle River, WI, USA) and a custom-written software (Rat-Rec Pro 5.0), processed by a fast Fourier transform and displayed as a color spectrogram.

Non-FM (“flat”) and FM 50-kHz USV calls were identified according to Brudzynski (2013); the latter category included all call types showing varying frequencies. In our lab, AMPH was consistently found not to affect the number of “flat” 50-kHz calls (Taracha et al., unpublished data). This finding is in line with a number (Ahrens et al. 2009; Pereira et al. 2014) while not all (e.g., see Simola et al. 2012; Wright et al. 2012, Supplementary material) of the relevant reports. Moreover, such calls consisted <1 % of all 50-kHz calls in our material, which observation is in accordance with the report of Maier et al. (2012). Hence, only FM 50-kHz calls were analyzed and are shown in this report.

### Preliminary USV testing and rat categorization, and TIPS-based preselection of rats

**Fig. 1:**
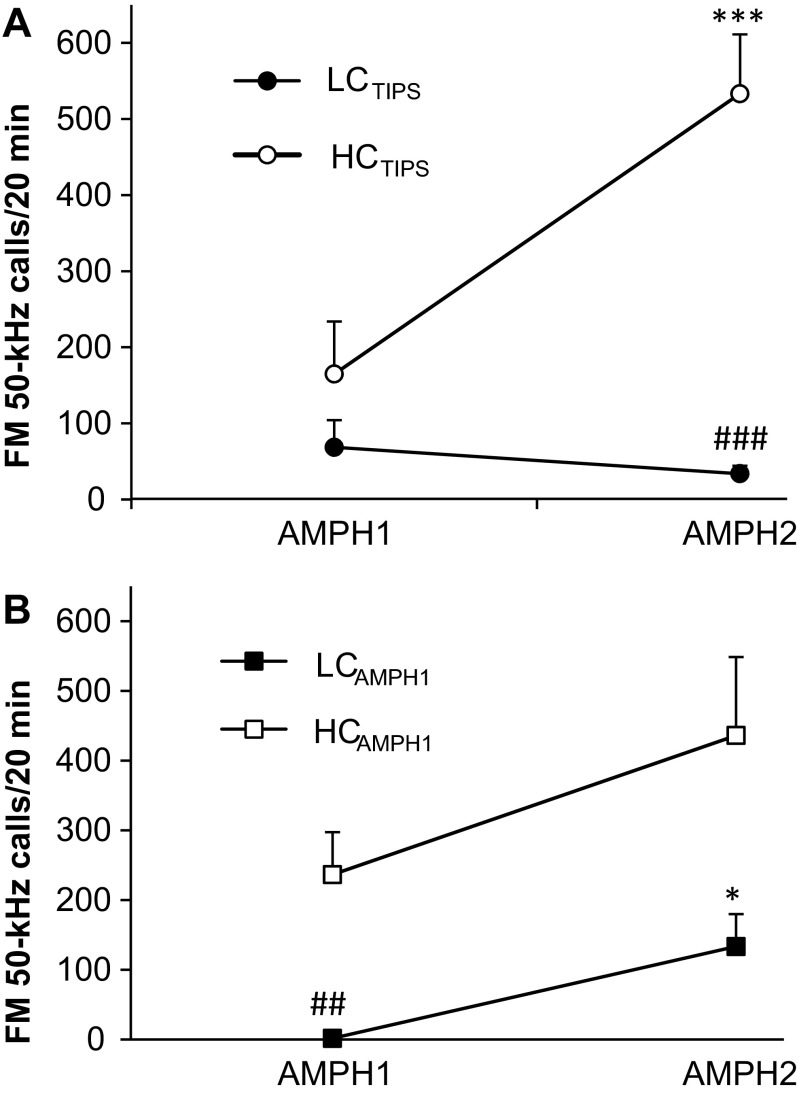
**a**, **b** Changes in the FM 50-kHz USV rate response to AMPH over the TIPS procedure in rats categorized by the sensitization of their FM 50-kHz USV rate response (panel **a**) or by their response to the first AMPH exposure (panel **b**). *p < 0.05, ***p < 0.001 vs. the respective value for AMPH1; ##*p* < 0.01, ###*p* < 0.001 vs. the corresponding value for the other rat subset

After acclimation and habituation (see the “Subjects” section above), the rats were subject to the TIPS procedure to create and identify rat subsets with diverging sensitization of their FM 50-kHz USV response to AMPH. This protocol was first used for locomotor sensitization of mice to morphine and cocaine (Valjent et al. 2010) and next was found effective for sensitization of rat 50-kHz USV response to AMPH (Taracha et al. 2012, 2014). USV testing was done as described in details elsewhere (Taracha et al. 2014, 2015). Briefly, the USV sessions took place in a room with ceiling and walls painted dull white and lit with incandescent matt white light bulbs. Two testing arenas (35.5 cm × 20 cm × 34 cm, L × W × H; with no bedding) were used concurrently; they were separated with a sound-attenuating wall and were thoroughly cleaned after each session. Each microphone was placed 35 cm above the bottom, centrally in relation to its assigned arena. The rats were given an ip AMPH dose (1.5 mg/kg) and then instantly tested for USV for 20 min. Six days later, all the rats were given an identical drug dose and were tested again for 20 min for their USV response. Next, the rats were classified as follows: the rats with the rise in their FM 50-kHz USV response (calls/20 min) to the second dose (AMPH2) as compared to that to the first dose (AMPH1) of >2 S.E.M. above the mean increase for the entire cohort were termed high-sensitized callers (HC_TIPS_, *N* = 10), and those with a change in their response to AMPH2 as compared to that to AMPH1 of >2 S.E.M. below the mean were termed low-sensitized callers (LC_TIPS_, *N* = 11). The remaining rats were excluded from further experimentation. A two-way ANOVA re-analysis of the USV data for the preselected rats has shown robust drug dose number and group effects and a strong drug dose number × group interaction effect (*F*_1,19_ = 17.1, *p* < 10^-3^; *F*_1,19_ = 25.1, *p* < 10^-3^, and *F*_1,19_ = 17.9, *p* < 10^-3^, respectively), see also Fig. 1a.

The preselected rats were also categorized by their USV response to AMPH1 using the so-called median split: the rats with the response below and above the median value were termed low callers (LC_AMPH1_) and high callers (HC_AMPH1_), respectively. For the odd total number of the preselected rats, the data for the rat with the median USV response were omitted from all analyses employing this categorization. A two-way ANOVA of the TIPS USV data for the LC_AMPH1_ and HC_AMPH1_ rats has yielded robust AMPH dose number (*F*_1,18_ = 9.11, *p* = 0.007) and group (*F*_1,18_ = 20.3, *p* < 10^-3^) effects, but no appreciable AMPH dose number × group interaction effect (*F*_1,18_ = 1.51, *p* = 0.23), see also Fig. 1b. A similar result was obtained for the entire starting rat cohort (*N* = 28) categorized by the median split (AMPH dose number effect: *F*_1,26_ = 18.7, *p* < 10^-3^; group effect: *F*_1,26_ = 31.0, *p* < 10^-3^; AMPH dose number × group interaction effect: *F*_1,26_ = 1.72, *p* = 0.20); data not shown. This similarity showed that the TIPS-based preselection did not considerably distort the composition of the study rats cohort in regard to their predilection to vocalize.

### Catheter implantation

The surgery was performed under general anesthesia achieved with an i.p. injection of a mixture of ketamine hydrochloride and xylazine (75 and 5 mg/kg b.w., respectively). A silastic catheter (0.625 mm o.d.) was inserted ∼35 mm deep into the right jugular vein through a small incision made directly right of the middle, at the neck level; the catheter position was secured with sutures to the neck muscles. The catheter’s distal end was subcutaneously threaded to exit the skin in the midscapular region, sutured to the underlying muscles, and closed with an obturator. After the surgery, the rats were housed singly and were allowed 6–7 days for recovery. The catheters were flushed daily with ∼0.2 ml of heparin and gentamycin sulfate solution (1.25 U/ml and 0.64 mg/ml, respectively) in Sal. The catheter patency was checked every 2–3 days in all rats (a few hours before consecutive training or extinction and before reinstatement session) by infusion of a short-acting barbiturate methohexital solution (10 mg/kg) that induces a brief loss of consciousness. Damaged or blocked catheters were replaced (on the day the problem was identified) with new catheters that were implanted into the left jugular vein or a femoral vein by the same procedure. Re-operated rats were allowed 2–3 days of recovery before re-entering the experimental paradigm. One rat that lost his catheter at the beginning of extinction course was excluded from further experimentation. The numbers of rats that underwent re-operation because of a problem with catheter were similar for the LC_TIPS_ and HC_TIPS_ rats (8 out of 11 and 7 out of 10, respectively), and only one rat in each of the subsets required one more catheter replacement (involving the femoral vein). The two rat subsets also contributed equally (50 %/50 %) to the group that did not require re-operation. Importantly, for the rats that did and did not require re-operation, there was a similar average “active” operandum nose-poking intensity for the entire self-administration training period of 44 “daily” sessions (608 and 619 nose-pokes/rat, respectively; see the next section for the relevant experimental design details). These numbers indicate that the catheterization-related surgeries did not considerably interfere with the instrumental response.

### AMPH self-administration training, extinction, and reinstatement

The equipment for drug self-administration training, extinction, and reinstatement was as previously described (Acewicz et al. 2012). The session start was signaled by turning on the conditioning chamber house light and an automated infusion of the priming AMPH dose or Sal. Nose-poking pre-defined number of times into the “active” operandum resulted in infusion of a pre-defined drug dose over 2 s, which was associated with a 2-s feedback tone and turning off of the house light for a 20-s time-out period. During time-out, the active operandum nose-pokes were not rewarded with infusions. The nose-pokes in the active operandum that did not trigger AMPH infusions, as well as all nose-pokes in the “inactive” operandum, were associated with a short sound (feedback noise) from the given operandum nose-poke counting circuit. Extinction sessions were run using the same scheme with Sal infusions. USV calls were collected using a microphone placed above the “inactive” operandum just below the chamber top. The centers of the active and inactive operanda were situated 16 cm apart.

**Fig. 2:**
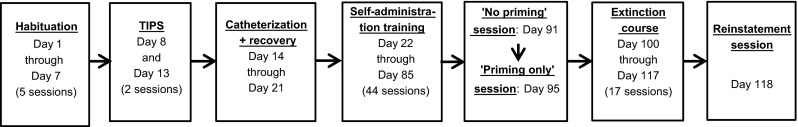
Scheme of experimental design

The subject rats were given 44 “daily” (except weekends) 2-h training sessions using an increasing fixed-ratio (FR-1 to FR-5) schedule of reinforcement. As low unitary drug doses reportedly better discriminate between rats differing in their acquisition of stimulant self-administration (Piazza et al. 1989, 2000; Klebaur et al. 2001; Mantsch et al. 2001; Granholm et al. 2015), training was started with a 0.03 mg/kg/infusion of AMPH. Because of poor self-administration acquisition, the dose was next changed to 0.06 mg/kg/infusion. Five days after completion of the training, the rats were given a single 40-min session (FR-5 with 0.06 mg/kg/infusion of AMPH) with no priming; 3 days later, they were given another 40-min session consisted of priming AMPH infusion (0.09 mg/kg) followed by earned Sal infusions (at FR-5). Next (after a 5-day break), the rats were given 17 daily 2-h extinction sessions using Sal at FR-5. Four LC_TIPS_ and five HC_TIPS_ rats (chosen randomly) received a NAC injection (90 mg/kg) 90 min prior to each extinction session; the other rats were given Sal instead. The choice of the NAC dose was based on earlier reports (Baker et al. 2003; Frankowska et al. 2014). One day after the 17th extinction session, all rats were evaluated for 2 h for self-administration reinstatement using AMPH priming of 0.06 mg/kg and earned Sal infusions (at FR-5) instead of 0.06 mg/kg infusions of AMPH. For a general scheme of the experimental design, please see Fig. 2.

Because of other projects that were running concurrently, USV was only recorded during selected sessions. Also, we did not register USV during the self-administration acquisition and extinction sessions performed immediately after weekend breaks because such breaks transiently interfere with the natural course of changes in the USV response to repeated psychostimulant treatment (Maier et al. 2012; Taracha et al. 2015).

### Statistical analysis

All data are expressed as the mean ± S.E.M. As nose-poking and USV data showed non-normal distributions, they were square root-transformed for statistical analyses and were next subject to a two-way or three-way ANOVA (with repeated measures on session) as required. Except when specified otherwise, the significance of between-group differences and within-group changes was tested with the Tukey test for unequal sample sizes. In all cases, a *p* < 0.05 was considered significant. All the analyses were run with the Statistica v. 8.0 software package (StatSoft, Tulsa, OK, USA).

## Results

### TIPS- versus AMPH1-based categorization: active operandum nose-poking during AMPH self-administration training

**Fig. 3:**
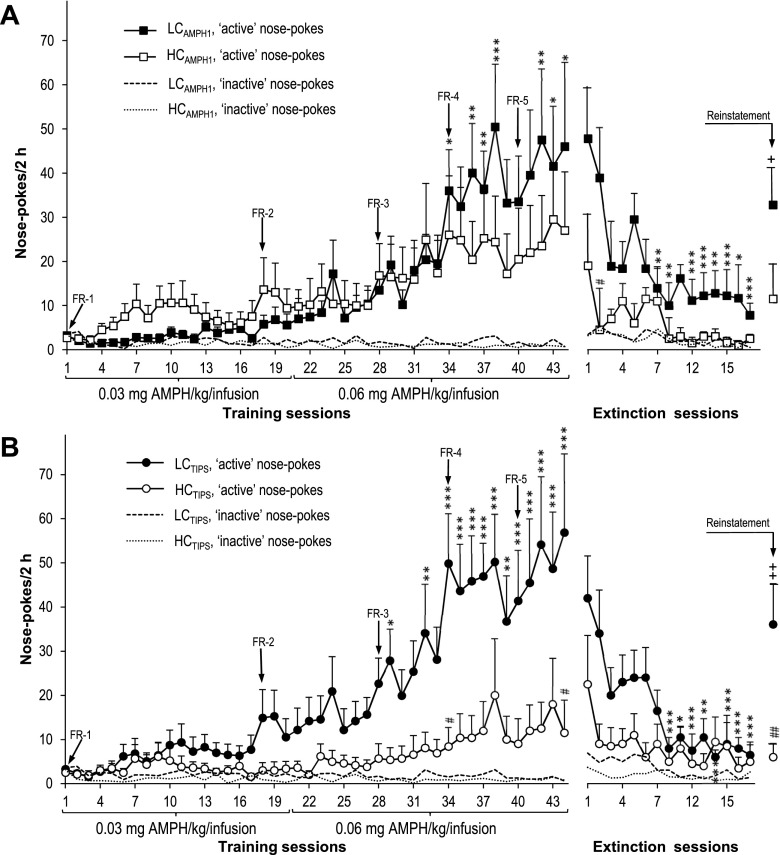
**a**, **b**. The effects of AMPH self-administration training, extinction, and reinstatement on nose-poking activity of the subject rats categorized by their FM 50-kHz USV response to the first drug exposure in the TIPS procedure (LC_AMPH1_ rats vs. HC_AMPH1_ rats, panel **a**) or by the sensitization of their FM 50-kHz USV response to the drug in the TIPS procedure (LC_TIPS_ rats vs. HC_TIPS_ rats, panel **b**). *Arrows* denote changes in the fixed ratio schedule of reinforcement. *p < 0.05, **p < 0.01, ***p < 0.001 vs. the respective value for the training or extinction session 1; #*p* < 0.05, ##*p* < 0.01 vs. the corresponding value for the other rat subset; +*p* < 0.05, ++*p* < 0.01 vs. the corresponding value for the last extinction session. Please note that the curves for inactive operandum nose-pokes are for illustration only, hence no error bars are shown

LC_AMPH1_ rats compared to HC_AMPH1_ rats tended to nose-poke slightly less during the first half of the training; an opposite tendency appeared during the second half and persisted until the end of the training (Fig. 3a). Two-way ANOVA yielded no significant group effect (*F*_1,18_ = 0.22, *p* = 0.65), but significant effects of session number and group × session number interaction (*F*_43,774_ = 7.06, *p* < 10^-3^, and *F*_43,774_ = 2.19, *p* < 10^-3^, respectively). Post-hoc test showed significant increases in nose-poking toward the end of the training period (at FR-4 and FR-5) in the LC_AMPH1_, but not in the HC_AMPH1_ rats. However, this difference did not translate into a significant difference between the two rat subsets during any training session.

LC_TIPS_ compared to HC_TIPS_ rats tended to nose-poke more after about 2 weeks; this difference increased steadily for the rest of the training (Fig. 3b). Two-way ANOVA yielded significant effects of group, session number, and group × session number interaction (*F*_1,19_ = 7.63, *p* = 0.012; *F*_43,817_ = 7.76, *p* < 10^-3^, and *F*_43,817_ = 4.46, *p* < 10^-3^, respectively). In the LC_TIPS_ rats, post-hoc test showed occasional significant increases in nose-poking at FR-3 (sessions 29 and 32) and stable and significant increases from training session 34 onward, i.e., starting with the first session at FR-4. No such effect was found in the HC_TIPS_ rats during any session. The main effect of group translated into a significant difference between the two subsets only for sessions 34 and 44 (FR-5).

**Fig. 4:**
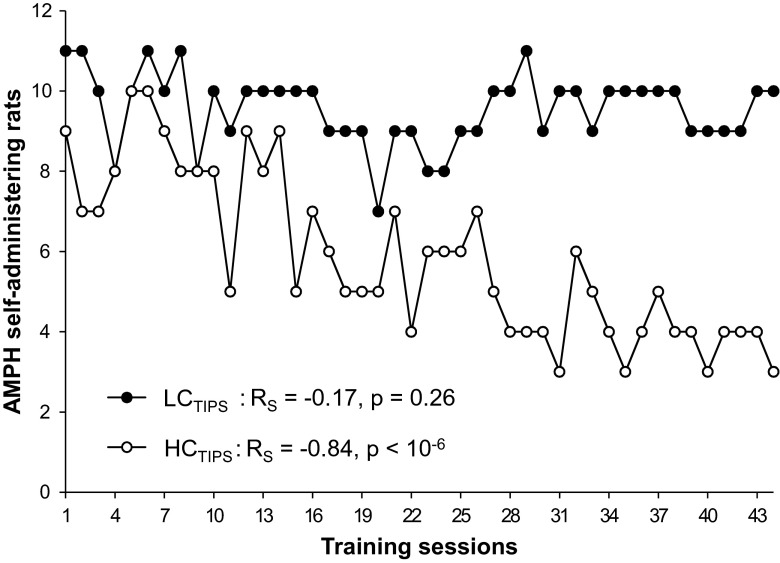
Changes in the numbers of AMPH self-administering rats in the rat subsets with high (HC_TIPS_) and low (LC_TIPS_) FM 50-kHz USV sensitization to AMPH throughout the course of self-administration training. *R*_s_—Spearman’s rank correlation coefficient

The number of AMPH self-administering LC_TIPS_ rats showed no substantial change during the training. In contrast, there was a major decline in the number of AMPH self-administering HC_TIPS_ rats that correlated significantly with training progression, see Fig. 4.

### TIPS- versus AMPH1-based categorization: active operandum nose-poking during self-administration extinction and reinstatement

A three-way ANOVA with LC_TIPS_/HC_TIPS_ status and NAC treatment as the between-subject factors showed a significant effect of extinction session number (*F*_14,224_ = 5.78, *p* < 10^-3^) and LC_TIPS_/HC_TIPS_ status (*F*_1,16_ = 4.67, *p* = 0.046), but no significant effect of NAC treatment (*F*_1,16_ = 2.37, *p* = 0.14) or of LC_TIPS_/HC_TIPS_ status × extinction session number, NAC treatment × LC_TIPS_/HC_TIPS_ status, NAC treatment × extinction session number, or NAC treatment × LC_TIPS_/HC_TIPS_ status × extinction session number interaction (*F*_14,224_ = 1.35, *p* = 0.18; *F*_1,16_ = 1.54, *p* = 0.23; *F*_14,224_ = 1.08, *p* = 0.38, and *F*_14,224_ = 0.65, *p* = 0.82, respectively). A three-way ANOVA with LC_AMPH1_/HC_AMPH1_ status and NAC treatment as the between-subject factors showed a significant effect of extinction session number (*F*_14,210_ = 5.44, *p* < 10^-3^), LC_AMPH1_/HC_AMPH1_ status (*F*_1,15_ = 10.83, *p* = 0.0050), NAC treatment (*F*_1,15_ = 4,95, *p* = 0.042), and LC_AMPH1_/HC_AMPH1_ status × extinction session number interaction (*F*_14,210_ = 1.75, *p* = 0.047), but no significant effect of NAC treatment × extinction session number or NAC treatment × LC_AMPH1_/HC_AMPH1_ status × extinction session number interaction (*F*_14,210_ = 1.15, *p* = 0.31, and *F*_14,210_ = 0.28, *p* = 0.996, respectively).

For both categorizations, there was a statistically significant decline in drug seeking in the HC rats but not in their LC counterparts over the course of extinction (see Fig. 3a, b, right panels). Neither the significant effects of the LC_TIPS_/HC_TIPS_ and LC_AMPH1_/HC_AMPH1_ statuses nor the significant NAC treatment effect for the LC_AMPH1_/HC_AMPH1_ status translated into a significant difference in nose-poking during any session. However, the residual nose-poking in the LC_AMPH1_ rats was not extinguished as fully as in the HC_AMPH1_ rats after the last extinction session (7.8 ± 4.9 vs. 2.5 ± 1.5, respectively).

The effects of AMPH reinstatement were analyzed using the nose-poking data from the last extinction session and the drug reinstatement session. A three-way ANOVA with NAC treatment and either the LC_TIPS_/HC_TIPS_ or LC_AMPH1_/HC_AMPH1_ status as the between-subject factors showed a significant effect of session (*F*_1,16_ = 9.84, *p* = 0.006, and *F*_1,15_ = 8.31, *p* = 0.011, respectively), a significant effect of the LC_TIPS_/HC_TIPS_ status, and a borderline significant effect of the LC_AMPH1_/HC_AMPH1_ status (*F*_1,16_ = 7.90, *p* = 0.013, and *F*_1,15_ = 4.53, *p* = 0.050, respectively). For either the LC_TIPS_/HC_TIPS_ or the LC_AMPH1_/HC_AMPH1_ status, there was no significant effect of NAC treatment (*F*_1,16_ = 0.74, *p* = 0.40, and *F*_1,15_ = 1.44, *p* = 0.25, respectively) or of session × NAC treatment (*F*_1,16_ = 0.53, *p* = 0.48, and *F*_1,15_ = 0.002, *p* = 0.98, respectively) or session × NAC treatment × LC/HC status interaction (*F*_1,16_ = 0.87, *p* = 0.36, and *F*_1,15_ = 0.48, *p* = 0.50, respectively). However, whereas there was no significant LC_AMPH1_/HC_AMPH1_ status × session interaction effect (*F*_1,15_ = 2.12, *p* = 0.17), there was a significant LC_TIPS_/HC_TIPS_ status × session interaction effect (*F*_1,16_ = 5.02, *p* = 0.040). Post-hoc test yielded a significant and robust instrumental reaction during the reinstatement session in both the LC_AMPH1_ and LC_TIPS_ rats, whereas no such reaction was found in either the HC_TIPS_ (p = 0.63) or the HC_AMPH1_ rats (*p* = 0.91); see Fig. 3a, b, right panels. However, the relative reinstatement-induced increases in the nose-poking from their respective extinction values were similar for the LC_AMPH1_ and HC_AMPH1_ rats (+321 vs. +360 %, respectively).

### FM 50-kHz USV during self-administration training, extinction, and reinstatement

**Fig. 5:**
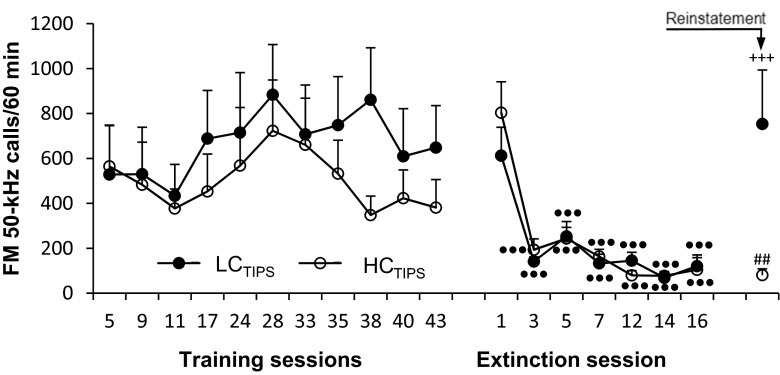
Changes in FM 50-kHz USV rate during AMPH self-administration training, self-administration extinction course, and drug reinstatement in rats categorized by the sensitization of their FM 50-kHz USV response to AMPH in the TIPS procedure. •••*p* < 0.001 vs. the respective value for extinction session 1, the Dunnett test. +++*p* < 0.001 vs. the respective value for extinction session 16, ##*p* < 0.01 vs. the respective value for the non-SA rats (the Tukey test for unequal sample sizes)

LC_TIPS_ and HC_TIPS_ rats did not significantly differ in the FM 50-kHz USV rate during AMPH self-administration training (see Fig. 5, left graph). Two-way ANOVA with LC_TIPS_/HC_TIPS_ status as the between-subject factor and session number as the within-subject factor yielded no significant effect of status (*F*_1,19_ = 0.52, *p* = 0.48), session number (*F*_10,190_ = 1.40, *p* = 0.18), or the interaction of status × session number (*F*_10,190_ = 0.51, *p* = 0.88).

During the course of AMPH self-administration extinction, the FM 50-kHz USV rate decreased dramatically in the initial phase of the course in both the LC_TIPS_ and HC_TIPS_ rats (see Fig. 5, right graph). A three-way ANOVA with repeated measures on extinction session number for rats categorized by NAC treatment and LC_TIPS_/HC_TIPS_ status yielded no sizable NAC treatment or LC_TIPS_/HC_TIPS_ status effect (*F*_1,16_ = 0.04, *p* = 0.84, and *F*_1,16_ = 0.03, *p* = 0.87, respectively) and no NAC treatment × LC_TIPS_/HC_TIPS_ status, NAC treatment × extinction session number, LC_TIPS_/HC_TIPS_ status × extinction session number, or NAC treatment × extinction session number × LC_TIPS_/HC_TIPS_ status interaction (*F*_1,16_ = 0.0022, *p* = 0.96; *F*_6,96_ = 0.33, *p* = 0.92; *F*_6,96_ = 0.73, *p* = 0.63, and *F*_6,96_ = 0.64, *p* = 0.70, respectively).

During reinstatement session, only the LC_TIPS_ rats showed an increase in their FM 50-kHz USV rate (see Fig. 5). A three-way ANOVA of the data from the last USV recording session during the course of self-administration extinction (extinction session 16) and from the reinstatement session, with repeated measures on session and LC_TIPS_/HC_TIPS_ status and NAC treatment as the between-subject factors showed a significant effect of session (*F*_1,16_ = 12.57, *p* = 0.0027), LC_TIPS_/HC_TIPS_ status (*F*_1,16_ = 6.60, *p* = 0.021) and session × LC_TIPS_/HC_TIPS_ status interaction (*F*_1,16_ = 12.26, *p* = 0.0030), but not of NAC treatment (*F*_1,16_ = 1.69, *p* = 0.21) or NAC treatment × LC_TIPS_/HC_TIPS_ status, NAC treatment × session, or NAC treatment × LC_TIPS_/HC_TIPS_ status × session interaction (*F*_1,16_ = 0.55, *p* = 0.48; *F*_1,16_ = 0.99, *p* = 0.33; and *F*_1,16_ = 0.46, *p* = 0.51, respectively). Post-hoc analysis yielded a significantly higher FM 50-kHz USV rate in the LC_TIPS_ compared to HC_TIPS_ rats during the reinstatement session.

### Effects of AMPH self-administration training, extinction, and reinstatement on FM 50-kHz USV and drug seeking: self-administering versus non-self-administering rats

To analyze the relationship between USV and acquisition of AMPH self-administration and drug seeking, the rats that had self-administered a total of no more than one drug infusion throughout the last seven sessions were termed non-SA rats (*N* = 7). The remaining rats (*N* = 14), which self-administered between 7.8 ± 1.9 and 13.4 ± 2.5 AMPH infusions/rat/session during each of those seven sessions, were termed SA rats. The average drug doses self-administered by the two subsets during that period are shown in Table 1.

**Table 1:** Voluntary AMPH intake during the last seven training sessions

Session number	AMPH dose [mg/kg]		Mann–Whitney *U* test *p*
	Non-SA rats (*N* = 7)	SA rats (*N* = 14)	
38	0.000 ± 0.000	0.806 ± 0.153	0.0002
39	0.000 ± 0.000	0.540 ± 0.121	0.0005
40	0.000 ± 0.000	0.467 ± 0.112	0.0011
41	0.000 ± 0.000	0.531 ± 0.134	0.0005
42	0.009 ± 0.009	0.613 ± 0.145	0.0007
43	0.000 ± 0.000	0.613 ± 0.128	0.0002
44	0.000 ± 0.000	0.634 ± 0.173	0.0005

#### Effects of AMPH self-administration training and special sessions

**Fig. 6:**
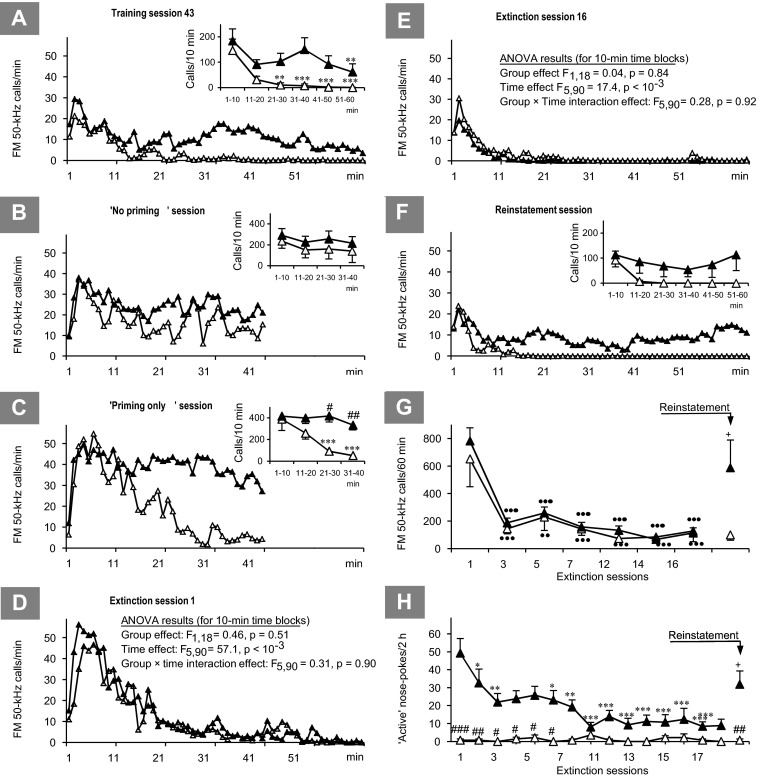
**a**–**h** Time profiles and summary values of the FM 50-kHz USV rate responses (graphs **a**–**g**) and instrumental responses (graph **h**) of non-SA rats (*empty triangles*) and SA rats (*filled triangles*) for the selected AMPH self-administration training sessions (graph **a**), special sessions (graphs **b** and **c**), selected self-administration extinction sessions (graphs **d**–**g**), and AMPH priming-induced reinstatement session (graphs **f**–**h**). *p < 0.05, **p < 0.01, ***p < 0.001 vs. the respective starting value; #*p* < 0.05, ##*p* < 0.01, ###*p* < 0.001 vs. the corresponding value for the other rat subset; +*p* < 0.05 vs. the corresponding value for the last extinction session (the Tukey test for unequal sample sizes). ••*p* < 0.01, •••*p* < 0.001 vs. the respective starting value (the Dunnett test)

At the end of session 43 (the last training session with USV recorded), the SA rats emitted considerable numbers of FM 50-kHz calls. In contrast, the FM 50-kHz USV rate in their non-SA counterparts during that session showed a rapid decrease to very low levels within the first 20 min and remained close to nil for the remainder of the 60-min USV recording period; see Fig. 6a. Two-way ANOVA with repeated measure on 10-min time blocks revealed significant effects of the SA/non-SA status and time block, and a tendency for SA/non-SA status × time block interaction (*F*_1,19_ = 6.25, *p* = 0.022; *F*_5,95_ = 10.8, *p* < 10^-3^, and *F*_5,95_ = 1.98, *p* = 0.088, respectively).

Omission of AMPH priming caused no notable difference in FM 50-kHz USV rate between the non-SA and SA rats, see Fig. 6b. A two-way ANOVA with repeated measures on 10-min time blocks yielded no significant effect of the SA/non-SA status, a significant effect of time block, and no SA/non-SA status × time block interaction (*F*_1,19_ = 1.37, *p* = 0.26; *F*_3,57_ = 3.74, *p* = 0.016, and *F*_3,57_ = 0.31, *p* = 0.82, respectively). However, the significant general time block effect did not translate into a significant change in either subset.

Interestingly, the special session consisted of “priming only” AMPH treatment produced highly diverging effects on FM 50-kHz USV rate in the non-SA and SA rats, see Fig. 6c; two-way ANOVA with repeated measures on 10-min time blocks yielded significant effects of both the SA/non-SA status, time block, and SA/non-SA status × time block interaction (*F*_1,19_ = 10.9, *p* = 0.0037; *F*_3,57_ = 17.2, *p* < 10^-3^, and *F*_3,57_ = 9.91, *p* < 10^-3^, respectively).

#### Effects of the course of AMPH self-administration extinction and AMPH reinstatement

During all extinction sessions, FM 50-kHz USV rate peaked similarly in the non-SA and SA rats after the first few minutes and rapidly declined thereafter. FM 50-kHz USV decreased radically after the first extinction session in all rats; similarly to the first session, a majority of residual calls occurred during the first 10 min of final extinction sessions (see Fig. 6d, e). Whereas non-SA rats compared to SA rats showed, in general, a weak FM 50-kHz USV throughout self-administration training (SA/non-SA status effect *F*_1,19_ = 7.04, *p* = 0.016; session number effect *F*_43,774_ = 7.06, *p* < 10^-3^; and group × session number interaction *F*_43,774_ = 2.19, *p* < 10^-3^; data not shown), there was no apparent difference in this behavior between the non-SA and SA rats at either the beginning or the end of the AMPH extinction course, see Fig. 6f, g. A three-way ANOVA for the total number of FM 50-kHz calls emitted during 60-min recording sessions for rats categorized by the SA/non-SA status showed a significant session number effect (*F*_6,96_ = 24.6, *p* < 10^-3^), but no significant effect of the SA/non-SA status (*F*_1,16_ = 0.77, *p* = 0.39) or NAC treatment (*F*_1,16_ = 0.15, *p* = 0.70) and no interaction of this treatment with either the SA/non-SA status (*F*_1,16_ = 0.16, *p* = 0.69) or extinction session number (*F*_6,96_ = 0.26, *p* = 0.96) or with both (*F*_6,96_ = 0.69, *p* = 0.66).

During AMPH reinstatement session, the changes in FM 50-kHz USV rate of the non-SA rats were quite uniform and very similar to those found during the 16th extinction session (cf. Figs. 6f, e). In contrast, 5 out of 14 SA rats showed a substantial increase in their FM 50-kHz USV rate throughout the reinstatement session, whereas the remaining SA rats showed a very low USV response. As a result of this heterogeneity, a two-way ANOVA with repeated measure on 10-min time blocks showed a significant effect of time block, but no significant effect of the SA/non-SA status or of SA/non-SA status × time block interaction (*F*_5,90_ = 5.32, *p* < 10^-3^, *F*_1,18_ = 2.69, *p* = 0.12, and *F*_5,90_ = 0.50, *p* = 0.77, respectively). However, the significant time block effect did not translate into a significant change in FM 50-kHz USV rate throughout this session in either rat subset; see Fig. 6f inset.

The effect of AMPH reinstatement was also tested using FM 50-kHz USV data from the latest available extinction session with recorded USV (session 16) and drug reinstatement session. A three-way ANOVA of the data for rats classified by the SA/non-SA status yielded a tendency for the SA/non-SA status effect (*F*_1,16_ = 3.43, *p* = 0.083), but no sizable effect of NAC treatment (*F*_1,16_ = 1.75, *p* = 0.21) or of NAC treatment × SA/non-SA status, NAC treatment × session, or NAC treatment × session × SA/non-SA status interaction (*F*_1,16_ = 0.82, *p* = 0.38; *F*_1,16_ = 1.29, *p* = 0.27; and *F*_1,16_ = 0.75, *p* = 0.40, respectively). However, there was a significant effect of session (*F*_1,16_ = 5.89, *p* = 0.027) and session × SA/non-SA status interaction (*F*_1,16_ = 5.33, *p* = 0.035). Post-hoc analysis showed a significant FM 50-kHz USV rate response to AMPH self-administration reinstatement in the SA rats only, see Fig. 6g.

#### Effects of AMPH self-administration extinction and reinstatement on drug seeking

The SA rats showed an intense nose-poking in the active operandum during the first session and a marked decrease in this activity during the next 6–8 extinction sessions (the data for sessions 8–9 were lost due to power outages), whereas the non-SA rats showed closed to nil active operandum nose-poking throughout the extinction course, see Fig. 6h. A three-way ANOVA with the SA/non-SA status and NAC treatment as the between-subject factors showed significant effects of the status and session number (*F*_1,16_ = 25.9, *p* < 10^-3^ and *F*_14,224_ = 3.28, *p* < 10^-3^, respectively), but not of NAC treatment (*F*_1,16_ = 0.00011, *p* = 0.98), and no sizable NAC treatment × SA/non-SA status, or NAC treatment × extinction session number, or NAC treatment × extinction session number × SA/non-SA status interaction (*F*_1,16_ = 0.74, *p* = 0.40; *F*_14,224_ = 0.60, *p* = 0.87; and *F*_14,224_ = 0.71, *p* = 0.76, respectively).

The effect of AMPH reinstatement was tested using nose-poking data from the last extinction session and drug reinstatement session. A three-way ANOVA of the data for rats classified by the SA/non-SA status yielded no significant effect of NAC treatment (*F*_1,16_ = 0.14, *p* = 0.72), or of NAC treatment × SA/non-SA status (*F*_1,16_ = 0.01, *p* = 0.92), NAC treatment × session (*F*_1,16_ = 1.03, *p* = 0.33), or NAC treatment × session × SA/non-SA status interaction (*F*_1,16_ = 0.51, *p* = 0.49). There was a significant effect of the SA/non-SA status and session (*F*_1,16_ = 17.90, *p* < 10^-3^ and *F*_1,16_ = 5.58, *p* = 0.031, respectively), and a near-significant session × SA/non-SA status interaction (*F*_1,16_ = 4.24, *p* = 0.056). Post-hoc test showed a significant relapse of instrumental reaction in the SA rats only, see Fig. 6h.

## Discussion

To the best of our knowledge, this is the first report to use 50-kHz USV during AMPH self-administration in rats. More importantly, this is the first study demonstrating an early prediction of a rat’s propensity for drug self-administration based on sensitization of the FM 50-kHz USV response to the drug. Notably, this potential was demonstrated with a relatively long (10-week) study period that extended well beyond the typical duration of studies utilizing the psychostimulant self-administration paradigm.

### Predictive value of 50-kHz USV-based rat categorizations

In most studies on the inter-individual differences in the 50-kHz USV response to addictive drugs, the subject rats were classified based on their response to the first drug dose (Burgdorf et al. 2007; Ahrens et al. 2013; Simola and Morelli 2015). That categorization allegedly reflects an individual’s ability to vocalize and sensitivity to the rewarding properties of the drugs (Simola and Morelli 2015). Our approach capitalizes on individual differences in the vulnerability to sensitization of the USV response to the subsequent drug exposures (Taracha et al. 2012, 2014, 2015). We believe this is a better way as it implicitly accounts for the underlying, albeit still unclear, neurobiological changes associated with progression toward drug addiction.

There was no statistically significant link between a rat’s FM 50-kHz USV response to the first AMPH exposure and its propensity for self-administering the drug. This finding is in line with the view that the rewarding action of a drug may initially encourage drug use, but it is not pivotal for addiction emergence (de Wit and Phillips 2012; Piazza and Deroche-Gamonet 2013). HC_AMPH1_ rats consumed more drug during the initial phase of the self-administration training; however, this difference vanished after a few weeks. Notably, whereas the HC_AMPH1_ rats continued to self-administer AMPH at a relatively constant rate during the final three training weeks, their LC_AMPH1_ counterparts steadily increased their drug intake, resulting in reversal of the drug intake ratio for the two subsets.

Although behavioral sensitization is absent from the addiction diagnostic criteria, it is often utilized as an index for evaluating the psychoactive action of addictive drugs (Strakowski and Sax 1998; Leyton 2007; Castner and Williams 2007; Taracha et al. 2012, 2014). Unexpectedly, beginning in the third week of self-administration training, LC_TIPS_ rats persistently self-administered AMPH several more times than HC_TIPS_ rats. However, there was no significant difference in the respective FM 50-kHz USV rates. The two groups seemed to titrate their drug intake to reach a desired pleasure level that did not much differ between them, as evidenced by their similar USV rates. This explanation is supported by the data showing an inverse relationship between voluntary drug intake and behavioral sensitization to the drug (Kamens et al. 2005; Scibelli et al. 2011; Ball and Slane 2014). These findings indicate the utility of poor sensitization to stimulants as a predictor of acquisition and reinstatement of self-administration of these drugs. They also suggest that sensitization may be protective against drug abuse. This observation is also in agreement with the hypothesis that sensitization of the mesolimbic reward system may lead to a compensatory reduction in the amount of self-administered drug (Darna et al. 2015). It is also in agreement with the idea that susceptibility to addictions is present in individuals with reward system dysfunction who then resort to addictive drugs to reach a satisfaction level that is unattainable with normal life activities (Vetulani 2001). The finding about sensitization may also be relevant to the blunted stimulant-induced striatal dopamine release in cocaine addicts compared to healthy controls (Narendran and Martinez 2008). In contrast to our findings, many studies have reported, as we mentioned in the Introduction, an association between behavioral sensitization to psychostimulants and facilitated acquisition of drug self-administration. This discrepancy is likely related to the fact that the latter association was observed in studies of unselected cohorts (e.g., see Vezina 2004), whereas the results of studies performed with consideration of the individual vulnerability to sensitization are in agreement with our findings.

According to the general multi-step theory of transition to addiction (Piazza and Deroche-Gamonet 2013), a necessary step in this process is a phase of intensified, sustained, and escalating drug use. The majority of our LC_TIPS_ rats conformed to this requirement, and only one of these rats (9 %) ceased voluntary AMPH intake during self-administration training. The HC_TIPS_ subset greatly differed in this regard; it showed a major (60 %) drop in the number of rats that self-administered drugs and the remaining self-administering rats lacked sizable escalation of drug intake. Notably, the HC_TIPS_ rats that ultimately stopped their voluntary AMPH intake (*N* = 6) compared to those that continued intake had very low drug seeking activity during the first 14 training sessions (1.3 ± 0.18 vs. 7.0 ± 3.6 active nose-pokes/rat/session).

Whatever doubt might remain about the relative predictive value of the two discussed USV-based categorizations for the self-administration paradigm, it would be resolved by a study with a longer training duration. An appropriate endpoint might be the transition from intensified, sustained drug use to the loss of drug intake control (Belin et al. 2009; Piazza and Deroche-Gamonet 2013; Deroche-Gamonet and Piazza 2014; Everitt 2014).

### Factors affecting FM 50-kHz USV in the drug self-administration training–extinction–reinstatement paradigm

Despite the growing use of 50-kHz USV in rodent studies of addiction, it remains unclear what this behavior actually reveals. Rodent USV seems to reflect emotional states that result from factors that are internalized and may not always be isolated (Barker et al. 2015). In an attempt to identify the link between the appetitive USV and potentially important factors for acquiring drug self-administration behaviors, we categorized the rats in terms of their voluntary AMPH intake at the end of self-administration training (SA/non-SA status).

A visual comparison of the FM 50-kHz USV and instrumental reaction data from the second-to-last (43rd) self-administration training session (Fig. 6a), first and second-to-last extinction sessions (Figs. 6d, e), and AMPH reinstatement session (Fig. 6f) did not reveal obvious links. Surprisingly, the rats vocalized more frequently during the first 10 min of these sessions irrespective of their SA/non-SA status. This indicates that the dominant effect on USV was exerted by the drug priming and context. The role of the latter, especially with familiar experimenter contact, might have been enhanced by the fact that the rats were individually housed after catheter implantation. As a result, they were deprived of direct contact with conspecifics. During the remaining training sessions, an appreciable FM 50-kHz USV was found in a significant minority (6 out of 14) of the SA rats, but it was not found in the non-SA rats. This difference indicates that the self-administered drug might have played a role in evoking the “late” vocalization. However, it might not be the only factor involved as the SA rats, in contrast to the non-SA rats, had no decrease in the FM 50-kHz USV rate throughout the “priming alone” session (Fig. 6c). No studied characteristics helped to identify this SA rat subset. In particular, there was no apparent link between the USV during the session and either the LC_TIPS_/HC_TIPS_ or LC_AMPH1_/HC_AMPH1_ status. The heterogeneity of the SA group with respect to FM 50-kHz USV reaction suggests dissimilarity in the changes occurring during prolonged AMPH self-administration. This diversification may be related to the decline in the rewarding properties of AMPH and/or progression toward full addiction. Some of the SA rats may already have been addicted to the drug at the end of the self-administration training period, which might have significantly changed the relationship between their FM 50-kHz USV and AMPH intake.

### Effect of NAC supplementation on the extinction and reinstatement of AMPH self-administration as well as on the corresponding FM 50-kHz USV rates

NAC has shown promise in the treatment of stimulant addiction, especially in rat cocaine self-administration models (Baker et al. 2003; Madayag et al. 2007; Amen et al. 2011; Murray et al. 2011; Ramirez-Niño et al. 2013; Frankowska et al. 2014). However, the data on its efficacy in drug addicts are equivocal (McClure et al. 2014; Asevedo et al. 2014), and studies on its potential utility in animal models of AMPH abuse are scarce. We previously found no effect of a 2-week NAC treatment period on the rewarding effects of intraperitoneal AMPH in LC_TIPS_ and HC_TIPS_ rats that were treated repeatedly with this stimulant (Taracha et al. 2015). The present study extended that finding to the AMPH self-administration training–extinction–reinstatement paradigm. Furthermore, the present study failed to show an effect of repeated NAC treatment on either the instrumental or USV response. In contrast to the earlier study, this failure cannot be attributed to the use of weak extinguishing methods or to comparisons of dissimilar, neurobiologically dissociable activities. Instead, the present data provide additional evidence that differences may be attributed to the differences between the mechanisms of action for AMPH and cocaine (Vanderschuren and Kalivas 2000; Williams and Undieh 2016), rendering NAC ineffective for AMPH abuse.

### Concluding remarks

The present results indicate that the FM 50-kHz USV intensity during AMPH self-administration training, extinction, and reinstatement depends, inter alia, on individual reactivity, the history and context of drug use, and the time that has elapsed since the last exposure. The effects of these factors can vary depending on the actual phase of this experimental paradigm. In contrast, but in agreement with the report by Barker et al. (2014), we found no link between the USV rate and key characteristics of psychostimulant self-administration, i.e., drug seeking behavior. This finding is in line with the similarity of 50-kHz USV in cocaine SA rats and their yoked partners (Maier et al. 2012). Hence, the factors that determine inter-individual differences in the acquisition of AMPH self-administration are not reflected by the FM 50-kHz USV emitted during the training, extinction, and reinstatement of this trait. Therefore, this USV does not seem useful for monitoring progression toward AMPH addiction in this experimental paradigm, which may be from both the related loss of drug rewarding action in the advanced stages of progression toward full addiction and involvement of a number of unidentified (environmental?) factors.

The most important finding in this study is that the sensitization of the FM 50-kHz USV response to AMPH in the TIPS procedure allows for identification of a major subset of rats with a high (≥90 %) likelihood of acquiring and maintaining AMPH self-administration. While the latter is not equal to the vulnerability to AMPH addiction, there is little if any doubt that the rats that eventually become addicted originate from the SA subset. Unexpectedly, this subset consisted of the poorly sensitized rats. In contrast, approximately 2/3 of the rats with high sensitization to the drug failed on the self-administration training. Hence, a poor sensitization to AMPH may be a risk factor for developing psychostimulant addiction.
